# Effects of *WNT1* c.110 T>C and c.505G>T mutations on osteoblast differentiation via the WNT1/β-catenin signaling pathway

**DOI:** 10.1186/s13018-021-02495-2

**Published:** 2021-06-02

**Authors:** Bashan Zhang, Rong Li, Wenfeng Wang, Xueming Zhou, Beijing Luo, Zinian Zhu, Xibo Zhang, Aijiao Ding

**Affiliations:** 1 Clinical Laboratory, Affiliated Dongguan People’s Hospital, Southern Medical University, No.3 Xinguchong Wandao South Road, Wangjiang District, Dongguan, 523059 China; 2 Department of Orthopedic, Affiliated Dongguan People’s Hospital, Southern Medical University, Dongguan, 523059 China

**Keywords:** Osteogenesis imperfecta, Osteoblast, *WNT1*, Mutation, β-Catenin

## Abstract

**Background:**

*WNT1* c.110 T>C and c.505G>T missense mutations have been identified in patients with osteogenesis imperfecta (OI). Whether these mutations affect osteoblast differentiation remains to be determined. This study aimed to investigate the effects of *WNT1* c.110 T>C and c.505G>T mutations on osteoblast function, gene expression, and pathways involved in OI.

**Methods:**

Empty vector (negative control), wild-type *WNT1*, *WNT1* c.110 T>C, *WNT1* c.505G>T, and *WNT1* c.884C>A (positive control) mutant plasmids were constructed and transfected into preosteoblast (MC3T3-E1) cells to investigate their effect on osteoblast differentiation. The expressions of osteoblast markers, including *BMP2*, *RANKL*, osteocalcin, and alkaline phosphatase (ALP), were determined using quantitative real-time polymerase chain reaction (RT-qPCR), western blotting (WB), enzyme-linked immunosorbent assay, and ALP staining assay, respectively. The mRNA and protein expression levels of *WNT1* or the expression levels of the relevant proteins involved in the WNT1/β-catenin signaling pathway were also determined using RT-qPCR, WB, and immunofluorescence (IF) assays after the different plasmids were transfected into MC3T3-E1 cells.

**Results:**

Compared with those in the wild-type group, in the mutation groups, the mRNA and protein expression levels of *BMP2* were suppressed, the expressions of osteocalcin and ALP were inhibited, and the mRNA and protein expression levels of *RANKL* were enhanced in MC3T3-E1 cells. WB and IF assays revealed that the protein expression levels of *WNT1* in MC3T3-E1 cells were downregulated in the mutation groups compared with those in the wild-type *WNT1* group. Furthermore, the expression levels of nonphosphorylated β-catenin (non-p-β-catenin) and phosphorylated GSK-3β (p-GSK-3β) were downregulated in the mutation groups compared with those in the wild-type group. However, no significant changes in the expression level of non-p-β-catenin or p-GSK-3β were observed in the mutation groups.

**Conclusions:**

*WNT1* c.110 T>C and c.505G>T mutations may alter the proliferation and osteogenic phenotype of MC3T3-E1 linked to the progression of OI via the inhibition of the WNT1/β-catenin signaling pathway. This is the first study to confirm the effect of *WNT1* c.110 T>C and c.505G>T missense mutations on osteoblast differentiation and propose a new molecular mechanism for OI development.

## Introduction

Osteogenesis imperfecta (OI) is a group of heritable connective tissue disorders characterized by increased bone fragility during early childhood, reduced bone mass, and frequent fractures [1–4]. It is currently believed that approximately 90% of OI cases are caused by autosomal dominant mutations in *COL1A1* and *COL1A2*. However, approximately 20–25% of patients with moderate-to-severe OI have pathogenic mutations in other genes [5]. Mutations such as *SERPINF1*, *P3H1*, *CRTAP*, *PPIB*, *BMP1*, *FKBP10*, *SP7*, *PLOD2*, *TMEM38B*, *PLOD2*, *P4HB*, *SPARC*, and *SEC24D* have been considered closely related to autosomal recessive OI cases [6]. Moreover, researches have shown that *WNT1* mutation affects osteoblast activity, leading to increased bone mass disorder, brittle fractures, and progressive bone abnormality in patients with OI [7, 8].

*WNT1*, a member of the WNT protein family, plays a vital role in regulating bone mass and maintains the homeostasis of bone metabolism. In vitro experiments have shown that *WNT1* defects can result in significantly decreased bone formation, whereas *WNT1* overexpression can promote bone formation [9]. In OI, studies have shown that biallelic mutations in *WNT1* result in recessive OI, whereas heterozygous mutations in *WNT1* are associated with early-onset osteoporosis in dominant hereditary families [10, 11]. More than 27 disease-causing *WNT1* mutations have been discovered thus far [12]. However, the biological functions in most of the *WNT1* mutations have not yet been elucidated.

The canonical WNT1 pathway initiates a signaling cascade by binding to the Frizzled and LRP5 receptors on the cell surface, resulting in the accumulation of nonphosphorylated β-catenin (non-p-β-catenin) in cells, and functions as a transcription factor to stimulate the transcription of the downstream target genes of the WNT signaling pathway [13]. A study has shown that *WNT1* mutations can affect the activation of the canonical WNT pathway and the mineralization of osteoblasts [11]. Another study has also shown that a missense mutation in exon 3 (c.505G>T) of *WNT1* resulted in the substitution of glycine by cysteine at position 169 (p.G169C); besides, the missense mutation in exon 2 (c.110G>T) of *WNT1* resulted in the conversion of isoleucine to threonine at position 37 (p.I37T) in patients with OI [6]. However, whether the mechanism of the *WNT1* c.110 T>C and c.505G>T mutations affect osteoblast differentiation remains to be determined.

For a better evaluation of the potential molecular mechanisms of *WNT1* mutations, a nonsense mutation (c.884C>A) was set as a positive control. This mutation causes the truncation of the last 76 amino acids of *WNT1*, which has been confirmed by western blotting (WB). Hence, c.884C>A expression can be detected at a position below the molecular weight of the wild-type *WNT1* [11]. Therefore, in this study, *WNT1* c.110 T>C, *WNT1* c.505G>T, and *WNT1* c.884C>A (positive control) mutant plasmids were constructed and transfected into osteoblasts to investigate the effects of the mutations on cell viability, expression levels of osteoblast markers, and activation of the WNT1/β-catenin pathway in MC3T3-E1 cells. This study showed the effect of WNT1 c.110 T>C and c.505G>T mutations on osteoblast differentiation for the first time and proposed a new molecular mechanism for the development of OI.

## Materials and methods

### Cell cultures

Preosteoblast (MC3T3-E1) cells (ATCC, VA, USA) were cultured in α-DMEM supplemented with 10% fetal bovine serum. Cells were cultured at 37 °C in an incubator under 5% CO_2_ and 90% humidity.

### Plasmid construction and transfection

Wild-type *WNT1*, *WNT1* c.110 T>C, *WNT1* c.505G>T, and *WNT1* c.884C>A mutant plasmids were synthesized by General Biosystems, Inc (Anhui, China). Empty plasmids were used as the negative control, and c.884C>A was used as a positive control. The MC3T3-E1 cells were grown for 24 h in 6-well plates with an initial cell density of 5 × 10^5^ cells/mL. Cells in each well were transfected with Vector, *WNT1*, c.110 T>C, c.505G>T, and c.884C>A using Lipofectamine 3000 (Invitrogen, CA, USA) according to the manufacturer’s instructions for subsequent quantitative real-time polymerase chain reaction (RT-qPCR) and WB experiments. MC3T3-E1 cell suspensions were transfected with different plasmids using an osteogenic differentiation medium (#MUXMT-90021, Cyagen Biosciences, Guangzhou, China). Cells were cultured in the osteogenic differentiation medium and seeded in coverslips of 6-well plates at a density of 2 × 10^4^ cells/mL, and then the plasmids were transfected into the MC3T3-E1 cells every 72 h for a total of 14 days. Samples were then collected for the enzyme-linked immunosorbent assay (ELISA) and alkaline phosphatase (ALP) staining assay. Three independent assays were performed.

### 3-(4,5-dimethylthiazol-2-yl)-5-(3-carboxymethoxyphenyl)-2-(4-sulfophenyl)-2H-tetrazolium (MTS) assay

Before transfection, cells were seeded into a 96-well plate for 24 h at a density of 1 × 10^4^ cells/well. Next, cells were transfected with empty vector, wild-type *WNT1*, *WNT1* c.110 T>C, *WNT1* c.505G>T, and *WNT1* c.884C>A mutant plasmids. After transfection, the cells were seeded in 96-well plates overnight, and then 100 μL of α-DMEM supplemented with 20 μL of CellTiter 96 AQueous One Solution Reagent (Promega, WI, USA) were added into the wells, which contained MTS and phenazine ethosulfate. The cell viability was determined at 492 nm using a 96-well plate reader (Bio-Rad Laboratories, CA, USA). Three independent assays were performed.

### ELISA and ALP activity assay

During the cultivation of MC3T3-E1 cells in the osteogenic differentiation medium for 14 days, the supernatants were collected for the ELISA experiments. The Mouse OT/BGP ELISA Kit (#CSB-E06917m) provided by Cusabio (Wuhan, China) was used. Briefly, cell culture media were added to 96-well plates, incubated with 100 μL of biotinylated antibodies for 60 min at room temperature, washed five times, incubated with 100 μL of HRP-conjugated streptavidin for 20 min at room temperature in the dark, incubated with 3,3′,5,5′-tetramethylbenzidine solution for 20 min, and incubated with the termination solution. The relative expression levels were then determined by measuring the absorbance at 450 nm. Three independent assays were performed.

Similarly, during the cultivation of cells in the osteogenic differentiation medium for 14 days, the cells were also collected and examined. Cells were stained with ALP according to the instructions of the BCIP/NBT Alkaline Phosphatase Color Development Kit (#C3206, Beyotime Biotechnology, Jiangsu, China). The coverslips were removed, washed twice with phosphate buffer saline, and fixed with 95% ethanol for 8 min. Subsequently, the cells were air-dried and incubated with a substrate solution at 37 °C for 30 min in the dark. After the reaction, the samples were washed with double-distilled water, counterstained with methyl green for 2 min, washed three times with double-distilled water, and air-dried. Five sections per sample were randomly selected for analysis under a microscope (OPTEC, TP510, Chongqing, China). Three independent assays were performed.

### RT-qPCR

Total RNA was isolated from the MC3T3-E1 cells using TRIzol reagent (Invitrogen) according to the standard protocol. Total RNA was reverse transcribed into cDNA using M-MLV Reverse Transcriptase (Promega, WI, USA) with random primers. *WNT1*, *BMP2*, and *RANKL* were amplified using SYBR Green Real-time PCR Master Mix (TOYOBO, Osaka, Japan) and specific primers as follows: *WNT1* forward, 5′-CGATGGTGGGGTATTGTGAAC-3′; *WNT1* reverse, 5′-CCGGATTTTGGCGTATCAGAC-3′; *BMP2* forward, 5′-GGGACCCGCTGTCTTCTAGT-3′; *BMP2* reverse, 5′-TCAACTCAAATTCGCTGAGGAC-3′; *RANKL* forward, 5′-AGGCTGGGCCAAGATCTCTA-3′; and *RANKL* reverse, 5′-GTCTGTAGGTACGCTTCCCG-3′. The relative expression levels of *WNT1*, *BMP2*, and *RANKL* were calculated using the 2^−ΔΔCT^ method. *GAPDH* was used as an internal control: *GAPDH* forward, 5′-ATCAAGTGGGGTGATGCTGG-3′, reverse, 5′-CCTGCTTCACCACCTTCTTGA-3′. Three independent assays were performed.

### WB

Cells were lysed using radioimmunoprecipitation assay buffer (Beyotime, Shanghai, China) supplemented with protease inhibitors and phosphatase inhibitors (Roche, Mannheim, Germany). Protein concentration was quantified using a Bradford kit (Pierce, IL, USA). Proteins (40 μg/sample) were separated on a 10% SDS-polyacrylamide gradient gel and transferred to PVDF membranes (Millipore, MA, USA). After blocking with 5% bovine serum albumin (BSA), the membranes were incubated with primary *WNT1* (ab15251, Abcam, USA), β-catenin (sc7199, Santa, USA), non-p-β-catenin (19807 T, Cell Signaling Technology, USA), GSK-3β (ab32391, Abcam, USA), p-GSK-3β (9323 s, Cell Signaling Technology), *BMP2* (9323 s, Proteintech, China), and *RANKL* (ab45039, Abcam, USA) antibodies and corresponding HRP-conjugated secondary antibodies. The blots were visualized using a chemiluminescence reagent (Millipore, CA, USA). The relative expressions of the proteins were normalized to that of *GADPH* (60004-1-lg, Proteintech, China) using Image-Pro software. Three independent assays were performed.

### Immunofluorescence (IF) assay

The cells cultured on the coverslips of 6-well plates were transfected with empty vector, wild-type *WNT1*, c.110 T>C, *WNT1* c.505G>T, and *WNT1* c.884C>A mutant plasmids. After 72 h of transfection, the cells were fixed with 4% paraformaldehyde, permeabilized with 0.5% Triton-100 (Calbiochem, CA, USA), and blocked with 5% BSA. Next, cells were incubated with *WNT1* (ab15251, Abcam), β-catenin (sc7199, Santa), non-p-β-catenin (19807 T, Cell Signaling Technology), GSK-3β (ab32391, Abcam), and p-GSK-3β (9323 s, Cell Signaling Technology) primary antibodies followed by FITC-conjugated secondary antibodies (Sangon, Shanghai, China). Positive signals were observed using a fluorescent microscope, and samples were analyzed using Image-Pro software. DNA was stained with 4′,6-diamidino-2-phenylinodole (Sigma-Aldrich, MO, USA) for 5 min. Three independent assays were performed.

### Statistical analysis

GraphPad Prism 7 was used for visualization. One-way analysis of variance followed by Tukey’s post hoc test for multiple comparisons was performed to compare differences between groups. P < 0.05 was considered to indicate a statistically significant difference.

## Results

### *WNT1* c.110 T>C and c.505G>T mutations affect MC3T3-E1 cell proliferation

**Fig. 1:**
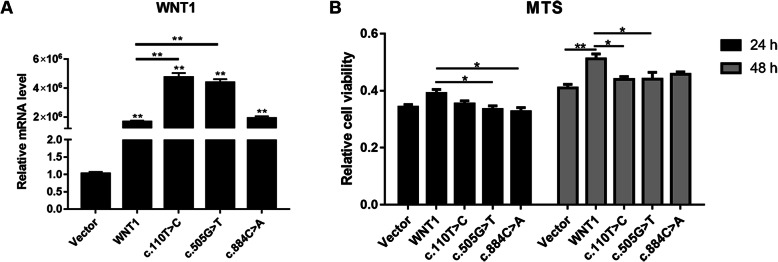
The situation of cell viability of MC3T3-E1 cells after transfection with empty vector, wild-type WNT1, WNT1 c.110 T>C, WNT1 c.505G>T, and WNT1 c.884C>A mutant plasmids at 24 and 48 h. **A** The mRNA expression levels of WNT1 after transfection at 24 h. **B** The viability of cells after transfection at 24 and 48 h. * *P* < 0.05 and ** *P* < 0.01 indicates significant differences

We transfected empty vector, wild-type *WNT1*, *WNT1* c.110 T>C, *WNT1* c.505G>T, and *WNT1* c.884C>A plasmids into MC3T3-E1 cells for 24 h individually, and then detected the expression levels of *WNT1*. The RT-qPCR results showed that the mRNA expression levels of *WNT1* were upregulated in the mutation and wild-type groups compared with those in the empty vector (Vector) group and that the mRNA levels of *WNT1* were downregulated in the *WNT1* c.110 T>C and c.505G>T mutation groups compared with those in the wild-type *WNT1* group, indicating that these plasmids were successfully transfected into the MC3T3-E1 cells and the *WNT1* mutations in MC3T3-E1 cells were successfully established (Fig. 1A). To investigate the effects of *WNT1* c.110 T>C and c.505G>T mutations on osteoblast proliferation, empty vector, wild-type *WNT1*, *WNT1* c.110 T>C, *WNT1* c.505G>T, and *WNT1* c.884C>A plasmids were transfected into the MC3T3-E1 cells for 24 and 48 h, respectively, and the cell viability in each group was measured using the MTS assay. Cell viability was found to be increased in the wild-type *WNT1* group compared with that in the Vector group. However, cell viability in the mutation groups was significantly lower than that in the wild-type *WNT1* group at 24 and 48 h, suggesting that *WNT1* mutations inhibited the viability of MC3T3-E1 cells (Fig. 1B).

### *WNT1* C.110 T>C and C.505G>T mutations affect the expression of osteoblast markers

**Fig. 2:**
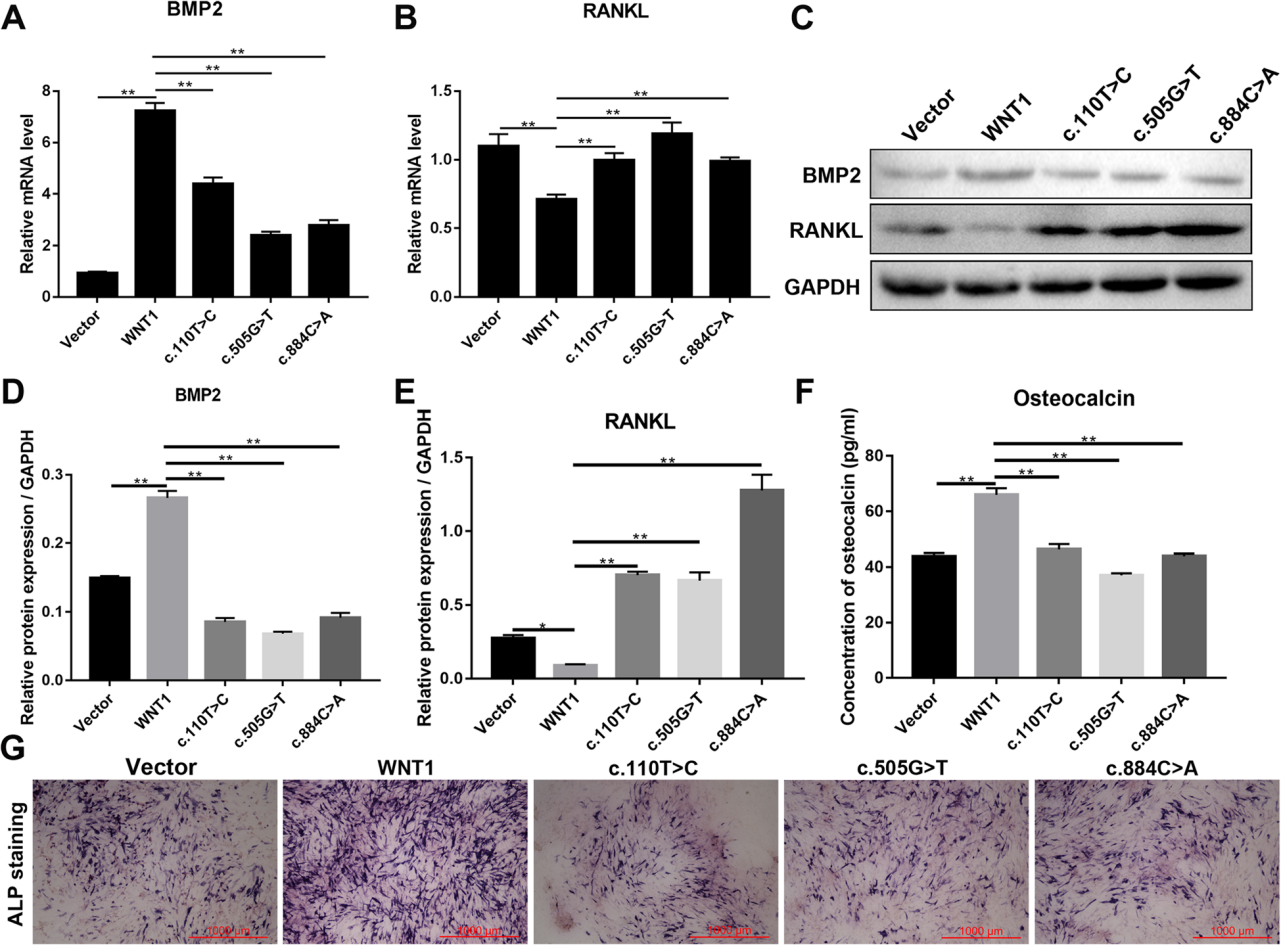
Effects of *WNT1* mutation on the mRNA and protein expression levels of *BMP2* (osteoblast marker) and *RANKL* (osteoclast marker) in the MC3T3-E1 cells. The mRNA expression levels of *BMP2* (**A**) and *RANKL* (**B**) were determined using quantitative real-time PCR. **C** The protein expression levels of *BMP2* and *RANKL* were determined using western blotting. **D**, **E** Semi-quantitative results of *BMP2* and *RANKL* proteins, respectively, using Image-Pro software. **F** Osteocalcin expression levels in the osteogenic differentiation medium on day 14 in different groups by ELISA. **G** Alkaline phosphatase expression levels in different groups by ALP staining. Bars represented 1000 μm * P < 0.05 and ** P < 0.01 indicate significant differences

For further investigation of the effects of *WNT1* c.110 T>C and c.505G>T mutations on osteoblast characteristics, the expression levels of the osteoblast differentiation marker *BMP2* and osteoclast differentiation marker *RANKL* were analyzed using RT-qPCR and WB in MC3T3-E1 cells transfected with empty vector, wild-type *WNT1*, *WNT1* c.110 T>C, *WNT1* c.505G>T, and *WNT1* c.884C>A plasmids. Compared with the Vector group, the cells transfected with wild-type *WNT1* plasmid had upregulated mRNA and protein expression levels of *BMP2* and downregulated mRNA and protein expression levels of *RANKL* (Fig. 2A–E). In addition, compared with the cells transfected with wild-type *WNT1* plasmid, the *WNT1* c.110 T>C, *WNT1* c.505G>T, and *WNT1* c.884C>A mutation groups showed downregulated mRNA and protein expression levels of *BMP2* and upregulated mRNA and protein expression levels of *RANKL* (Fig. 2A–E). The ELISA results showed that after induction for 14 days, the content of osteocalcin in the osteogenic differentiation medium in the wild-type *WNT1* group was significantly higher than that in the Vector group and also higher than that in the mutation groups (Fig. 2F). The ALP staining results showed that the ALP content in the osteogenic differentiation medium in the wild-type *WNT1* group was significantly higher than that in the Vector group and also higher than that in the mutation groups (Fig. 2G), suggesting that the c.110 T>C and c.505G>T mutation sites of *WNT1* inhibited the expression of osteocalcin and ALP during osteogenic induction and affected the osteogenic differentiation of the cells.

### *WNT1* c.110 T>C and c.505G>T mutations affect the WNT1/β-catenin signaling pathway

**Fig. 3:**
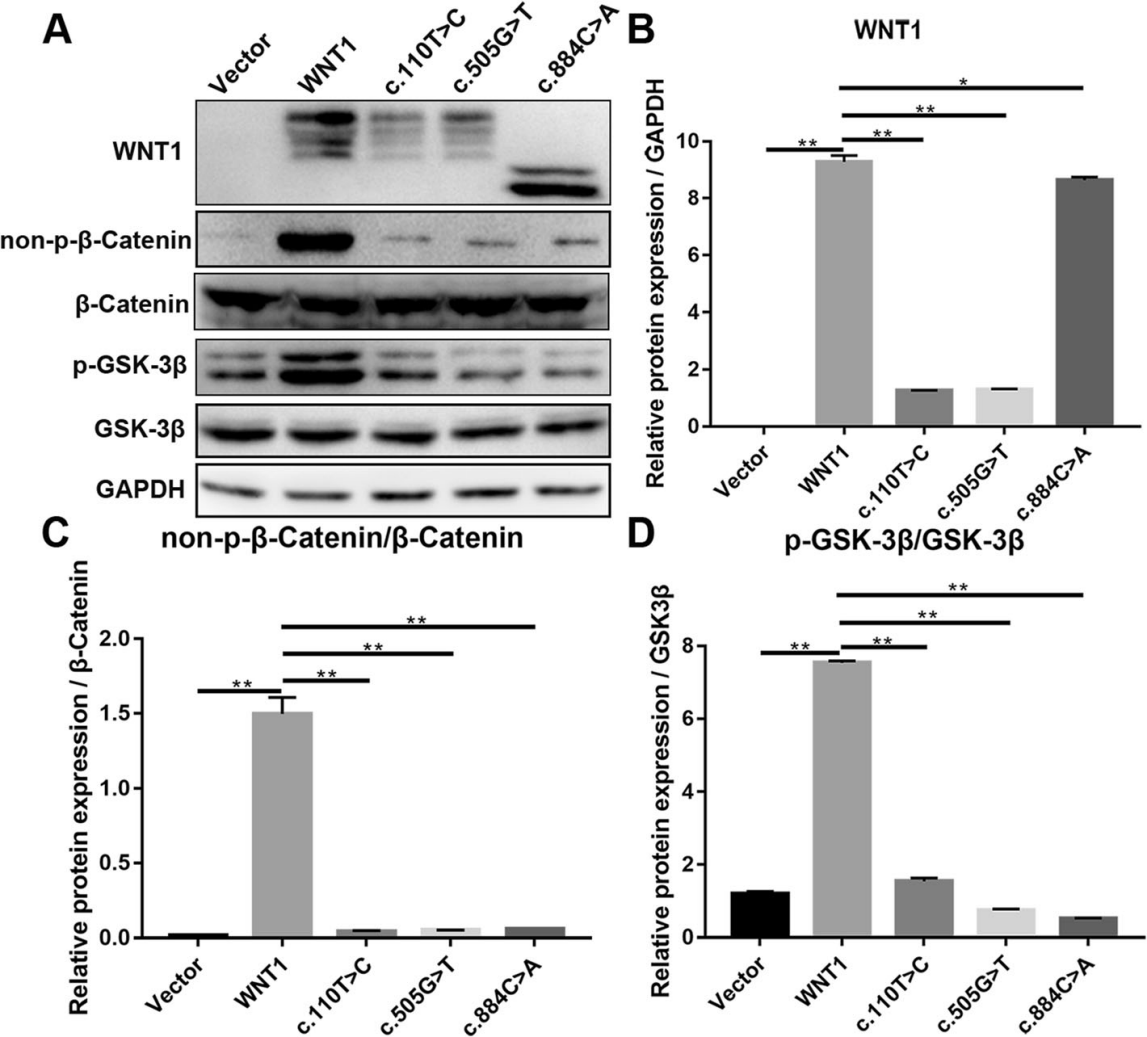
The protein expression levels of *WNT1* and important factors related to the WNT1/β-catenin signaling pathway in cells transfected with *WNT1* wild-type and mutant plasmids. **A** The protein expression levels of *WNT1* and other important factors related to the WNT1/β-catenin signaling pathway in the cells. **B**–**D** Semi-quantitative analysis of protein levels in graph A. * P < 0.05 and ** P < 0.01 indicate significant differences

**Fig. 4:**
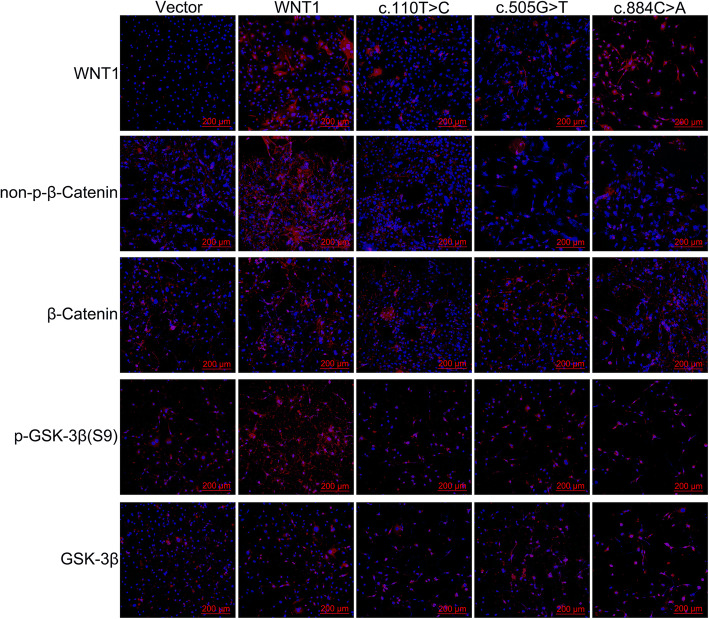
Protein expression levels of *WNT1* and essential proteins related to the WNT1/β-catenin signaling pathway detected using IF. Blue represents the nucleus and red represents the protein. Bars denote 200 μm

For further evaluation of *WNT1* expression in the mutation and wild-type groups and the possible mechanism underlying the effects of *WNT1* mutations on the differentiation of osteoblasts, MC3T3-E1 cells were transfected with *WNT1* and c.110 T>C, c.505G>T, and c.884C>A plasmids. Next, the expression levels of proteins related to the WNT1/β-catenin signaling pathway were validated in transiently transfected cells. WB and IF assays revealed that wild-type *WNT1* plasmids were able to upregulate *WNT1* expression at the protein level in MC3T3-E1 cells compared with that in the Vector group, but mutation plasmids downregulated *WNT1* expression at the protein level in MC3T3-E1 cells compared with that in the wild-type *WNT1* group. The highest expression occurred in the wild-type cells (Figs. 3A, B; 4; and 5A). Compared with wild-type *WNT1* cells, cells transfected with *WNT1* c.110 T>C, *WNT1* c.505G>T, and *WNT1* c.884C>A mutant plasmids showed downregulated expressions of non-p-β-catenin and p-GSK-3β (Figs. 3C, D; 4; and 5B, C), demonstrating that *WNT1* c.110 T>C and c.505G>T mutations might inhibit osteoblast proliferation and the expression of *BMP2* by weakening the WNT1/β-catenin signaling pathway.

**Fig. 5:**
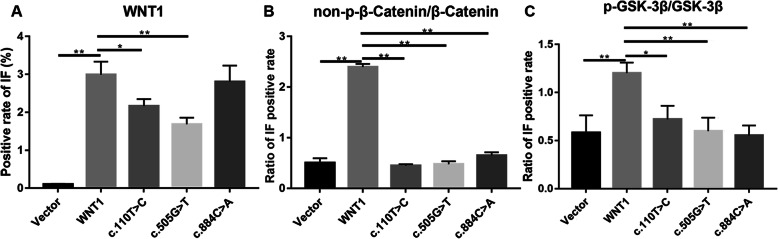
Semi-quantitative results of IF for the *WNT1*, non-p-β-catenin, and p-GSK-3β protein expression levels in Fig. 4. * P < 0.05 and ** P < 0.01 indicate significant differences

## Discussion

In a previous study, four new heterozygous *WNT1* mutations (c.110 T>C, c.505G>T, c.385G>A, and c.506G>A) were found to be associated with OI in four independent pedigree peripheral blood samples [6]. Of these, a missense mutation (*WNT1* c.110 T>C) located in exon 2 caused the conversion of isoleucine to threonine. Further, a missense mutation in exon 3 (*WNT1* c.505G>T) allowed the substitution of glycine by cysteine. However, currently, the physiological significance of these mutations remains unclear. Therefore, in the present study, we specifically analyzed the effects of *WNT1* c.110G>T and c.505G>T mutations on osteoblast differentiation in MC3T3-E1 cells for the first time. The results revealed that *WNT1* c.110 T>C and c.505G>T mutations had effects on osteoblast proliferation and the WNT1/β-catenin signaling pathway. Wild-type *WNT1* could induce the expression of osteoblast differentiation markers such as *BMP2*, osteocalcin, and ALP; suppress the expression of the osteoclast differentiation marker *RANKL*; and activate the WNT1/β-catenin signaling pathway; however, these effects were considerably impaired in the presence of the *WNT1* c.110 T>C and c.505G>T mutations.

In healthy humans, bone resorption of osteoclasts and bone formation of osteoblasts contribute to the maintenance of homeostasis. The disruption of the balance between bone resorption and bone formation of osteoblasts leads to the development of bone diseases such as osteoporosis, which is a critical feature in patients with OI [14, 15]. Research has been shown that *BMP2* and *RANKL* induce osteoblast and osteoclast differentiation, respectively [16–20], and that osteoblast cell viability in *WNT1*-deficient mice is reduced and associated with the fracture phenotype [21]. In this regard, our study found that *WNT1* c.110G>T and c.505G>T mutations inhibited the cell viability, weakened the mRNA and protein expression levels of *BMP2*, and enhanced the mRNA and protein expression levels of *RANKL*, indicating that *WNT1* c.110G>T and c.505G>T mutations can lead to osteoblast phenotype dysplasia, inhibit bone formation, and easily lead to an increased risk of osteoporosis and fracture.

A study has also revealed that *WNT1* mutations could lead to changes in the bone structure by deactivating the canonical WNT pathway [12]. Although some mutant forms have induced the activation of the *WNT1* signaling pathway, the expressions of the downstream target genes of the WNT signaling pathways are dysregulated and osteoblast mineralization is impaired [11]. The canonical WNT/β-catenin signaling pathway is activated by a combination of WNT and the Frizzled/LRP5/6 complex, which mediates the activation of β-catenin and then activates downstream gene expression. Furthermore, a functional study of essential receptors in the WNT signaling pathway has revealed that loss-of-function mutations in LRP5 can lead to reduced bone formation and decreased bone mass [22]. However, when there is no extracellular WNT, GSK-3β adds a phosphate group to β-catenin, resulting in the hydrolysis of β-catenin, decreased expression of β-catenin in cells and, ultimately, inhibition of the WNT/β-catenin signaling pathway. When the WNT signal is present, GSK-3β-mediated β-catenin hydrolysis is inhibited and downstream target genes of β-catenin can be activated [23].

Thus, the present study evaluated the effects of c.110G>T and c.505G>T mutations on *WNT1* expression. The WB results revealed that the *WNT1* c.110G>T and c.505G>T mutations decreased the levels of p-GSK-3β and non-p-β-catenin compared with that in the wild-type *WNT1* group, suggesting that *WNT1* c.110G>T and c.505G>T mutations are associated with decreased *WNT1* activation and decreased activation of the canonical WNT1/β-catenin signaling pathway. However, the precise mechanisms require further study for better understanding.

## Conclusion

This study is the first to confirm the effect of *WNT1* c.110 T>C and c.505G>T mutations on osteoblast differentiation via the WNT1/β-catenin signaling pathway, and consequently, a possible cause of OI at the cellular level. These findings suggest a new molecular mechanism for OI development.

## Data Availability

The datasets used and/or analyzed in the current study are available from the corresponding author on reasonable request.
